# Modulation of gastrointestinal motility beyond metoclopramide and domperidone: Pharmacological and clinical evidence for phytotherapy in functional gastrointestinal disorders

**DOI:** 10.1007/s10354-017-0557-3

**Published:** 2017-04-19

**Authors:** Ahmed Madisch, Bettina R. Vinson, Heba Abdel-Aziz, Olaf Kelber, Karen Nieber, Karin Kraft, Martin Storr

**Affiliations:** 1 Gastroenterologie, Interventionelle Endoskopie, Diabetologie, KRH Klinikum Siloah, Hannover, Germany; 2 Medical and Clinical Affairs Phytomedicines, Innovation and Development, Phytomedicines Supply and Development Center, Bayer Consumer Health, Steigerwald Arzneimittelwerk GmbH, Darmstadt, Germany; 3 Scientific Strategy Phytomedicines, Innovation and Development, Phytomedicines Supply and Development Center, Bayer Consumer Health, Steigerwald Arzneimittelwerk GmbH, Darmstadt, Germany; 4 Institut für Pharmazie, Universität Leipzig, Leipzig, Germany; 5 Lehrstuhl für Naturheilkunde, Zentrum für Innere Medizin, Universitätsmedizin Rostock, Rostock, Germany; 6 Zentrum für Endoskopie, Oßwaldstraße 1, 82319 Starnberg, Germany

**Keywords:** Irritable bowel syndrome, Functional dyspepsia, Prokinetic drugs, Herbal medicinal product, Evidence-based medicine, Reizdarmsyndrom, Funktionelle Dyspepsie, Prokinetika, Phytopharmakon, Evidenzbasierte Medizin

## Abstract

The prokinetic cisapride, an important therapeutic option in functional gastrointestinal (GI) disorders, was withdrawn from the market 15 years ago due to rare severe side effects. Likewise in 2014, the use of metoclopramide (MCP) and domperidone in functional GI disorders (FGID) was restricted, consequently leaving a therapeutic gap in clinical practice. A systematic review revealed that the herbal medicinal product (HMP) STW 5 presents a therapeutic option equivalent to MCP and cisapride. STW 5 is the only HMP for which efficacy has been shown in randomized controlled clinical trials (RCTs) in functional dyspepsia and irritable bowel syndrome, based on its multitarget effect on numerous etiological factors. Due to an outstanding favorable safety profile, STW 5 allows an effective and safe use in FGID without a limitation of the duration of the treatment.

## Zusammenfassung

Nachdem das Prokinetikum Cisaprid, eine wichtige Therapieoption bei funktionellen Magen-Darm-Erkrankungen, bereits im Jahr 2000 in Deutschland aufgrund seltener schwerer Nebenwirkungen vom Markt genommen wurde, wurden im Jahr 2014 auch Metoclopramid (MCP) und Domperidon von der Anwendung bei funktionellen gastrointestinalen Erkrankungen ausgeschlossen. Diese Entscheidungen hinterlassen eine therapeutische Lücke. Durch ein systematisches Review wurde das pflanzliche Arzneimittel STW 5 als eine zu MCP und Cisaprid gleichwertige Therapieoption identifiziert. STW 5 ist dabei das einzige pflanzliche Arzneimittel, für das die Wirksamkeit sowohl bei der funktionellen Dyspepsie als auch beim Reizdarmsyndrom in randomisierten klinischen Doppelblindstudien dokumentiert ist. Grundlage dafür ist seine Multi-Target-Wirkung auf ätiologische Faktoren der funktionellen gastrointestinalen Erkrankungen. Aufgrund seines sehr günstigen Sicherheitsprofils ist mit STW 5 eine wirksame und nebenwirkungsarme Behandlung möglich, ohne dass die Anwendungsdauer begrenzt ist.

## Introduction

Prokinetic drugs such as metoclopramide (MCP) and domperidone were important drugs in the treatment of functional gastrointestinal disorders (FGID). However, this prokinetic approach was questioned when the prokinetic drug cisapride, formerly a standard treatment, was withdrawn from the market in 2000 due to rare cardiac side effects.

In 2014, new restrictions were released by the European Medicines Agency (EMA) and numerous national regulatory authorities such as the Austrian AGES or the German BfArM, after FDA had been issuing a black box warning restricting the use of MCP due to the risk of rare extrapyramidal side effects.

Within the European Union, MCP is presently no longer authorized for the treatment of chronic conditions such as dyspepsia and gastroesophageal reflux diseases. The same applies to domperidone, due to rare cardiac side effects.

This requires revising clinical data in order to identify alternative treatments for these diseases. Associated with a long history, especially herbal medicinal products (HMPs) are widely used for FGID including functional dyspepsia (FD) and irritable bowel syndrome (IBS), with a proven favorable safety profile. The question is whether there are HMPs available for which, on the one hand, therapeutic equivalence to MCP, domperidone, or cisapride has been shown, and which, on the other hand, are likewise effective in both FD and IBS, as efficacy in both syndromes is, due to their frequent overlap, an important therapeutic advantage. In addition, these products should have a well-proven and a favorable safety profile.

## Materials and methods

**Fig. 1:**
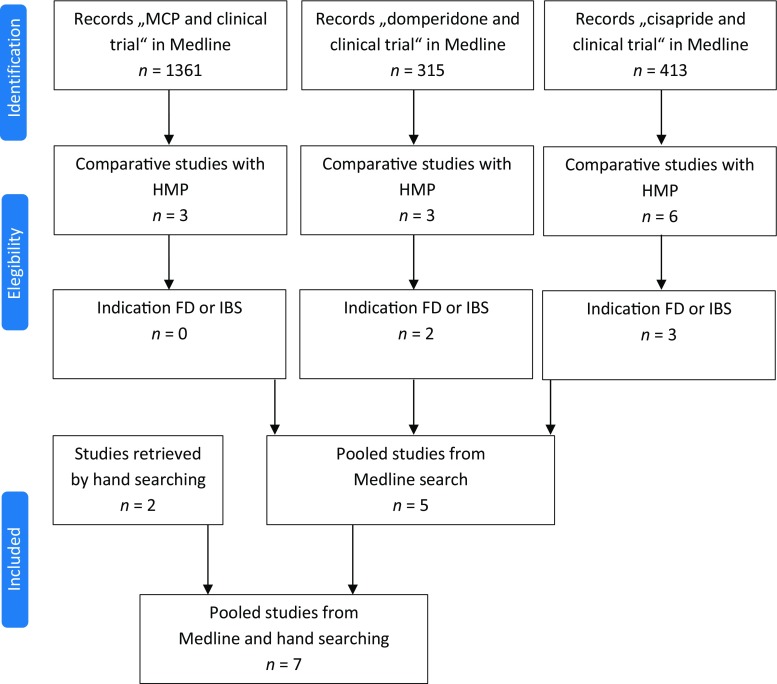
Flow diagram of the literature search for identifying clinical studies comparing prokinetics with herbal medicinal products (*HMP*). Medline search was conducted via PubMed (November 2016). *MCP* metoclopramide, *FD* functional dysfunction, *IBS* irritable bowel syndrome

To find such treatments, a systematic literature review was conducted, in accordance with the PRISMA statement, to identify HMPs, for which a therapeutic efficacy comparable to MCP, domperidone, or cisapride in FD or IBS has been shown (Fig. 1). A database search was conducted in PubMed, limited to clinical trials, with the terms metoclopramide, domperidone, or cisapride. As herbal medicinal products cannot be reliably retrieved by a MeSH term, the hits were analyzed by hand, and all clinical studies comparing one of these three prokinetic drugs with a HMP in the indications FD or IBS were identified. The search was complemented by cross referencing and hand searching to assure completeness.

In a further step, substances identified by the literature review were checked to determine whether there are clinical trials showing efficacy in both FD and IBS. Given this, the efficacy and the safety profile of the respective HMP, which is also based on postmarketing surveillance data and the mechanisms of action were presented, in comparison to those of the prokinetics.

## Results

As is known, the vast majority of patients with FD also has IBS [1]. Therefore, an effective treatment for FD suitable to replace the prokinetics should have clinically proven efficacy in both FD and IBS. Thus, the comparability to prokinetics alone is not sufficient to qualify a medicinal product as suitable to close the therapeutic gap left by these products. Also the efficacy in IBS needs to be proven.

## Evaluation of comparison studies of prokinetics and HMPs: clinical studies for FD and IBS

Seven comparative studies showing equivalence of HMPs with MCP, domperidone, or cisapride were identified via Medline (Fig. 1). In three studies, the HMP tested was a herbal combination medicine, STW 5 (Iberogast®). One study was identified for STW 5-II, a research combination.

One study each was also identified for a combination preparation from caraway oil and peppermint oil, for the Japanese combination preparation rikkunshito, and for the Chinese combinations hewei xiaopi capsule and sinisan (Table 1). For these three latter products the additional efficacy in IBS is not proven; therefore, they have not been pursued further in this review.

**Table 1:** Studies comparing STW 5 and prokinetics

Medicinal products studied	Study type	Study characteristics	Jadad score of trial quality	HMP with RCTs in FD and IBS	Ref
STW 5, metoclopramide	Randomized controlled, single blind study in FGID	Multicenter single-blinded clinical trial in 77 patients with functional gastroenteropathy. Metoclopramide liquid vs. STW 5 liquid, 2 weeks. End points: GI symptoms including fullness, stomach cramps and heartburn; tolerability	2	Yes^a^	[3]
STW 5, metoclopramide	Retrospective epidemiological cohort study in FD	Multicenter, retrospective, pharmacoepidemiological cohort study in 961 patients with functional dyspepsia. End points: Number of symptom free patients after treatment, days of inability to work	n.a.	Yes^a^	[4]
STW 5, STW 5-II, cisapride	Double-blind, randomized controlled clinical trial in FD	Randomized controlled clinical trial in 183 patients with dysmotility type of functional dyspepsia. Double-dummy design. 4 weeks treatment, 6 months follow-up. End point: gastrointestinal symptom score (GIS) [5]	5	Yes^a^	[6]
Combination of peppermint and caraway oil, cisapride	Double-blind, randomized controlled clinical trial in FD	Randomized controlled clinical trial in 120 patients with functional dyspepsia. Double-dummy design. 4 weeks treatment. End point: pain score	5	No	[31]
Rikkunshito, domperidone	Open, randomized trial in FD	Open clinical trial in 27 patients over 4 weeks. End point gastrointestinal symptom rating scale (GSRS)	2	No	[32]
Hewei xiaopi capsules, domperidone	Open, randomized trial in FD	Open clinical trial in 63 patients over 4 weeks. End point: FD symptoms	1	No	[33]
Sinisan (modified), cisapride	Open, randomized trial, IBS	Open clinical trial in 47 patients over 8 weeks. End point: IBS symptom scoring	1	No	[34]

^a^ For STW 5 only, RCS supporting both efficacy in FD and IBS and post marketing surveillance safety are data published [10]. Jadad score [35] for clinical trials: ≥3 good quality; *n.a.* not applicable, *MCP* metoclopramide, *HMP *herbal medicinal product, *FD* functional dysfunction, *IBS* irritable bowel syndrome

Searching for routine postmarketing surveillance data on safety in a next step, such data could be identified only for STW 5, but not for STW 5-II; thus, the presentation of data on safety and efficacy, on the mechanisms of action, focuses on STW 5.

## Clinical efficacy and safety

For STW 5, a herbal combination containing, among other herbal drug extracts, the fresh herbal extract of bitter candytuft (*Iberis amara* L) [2], three studies on FGID were identified.

**Fig. 2:**
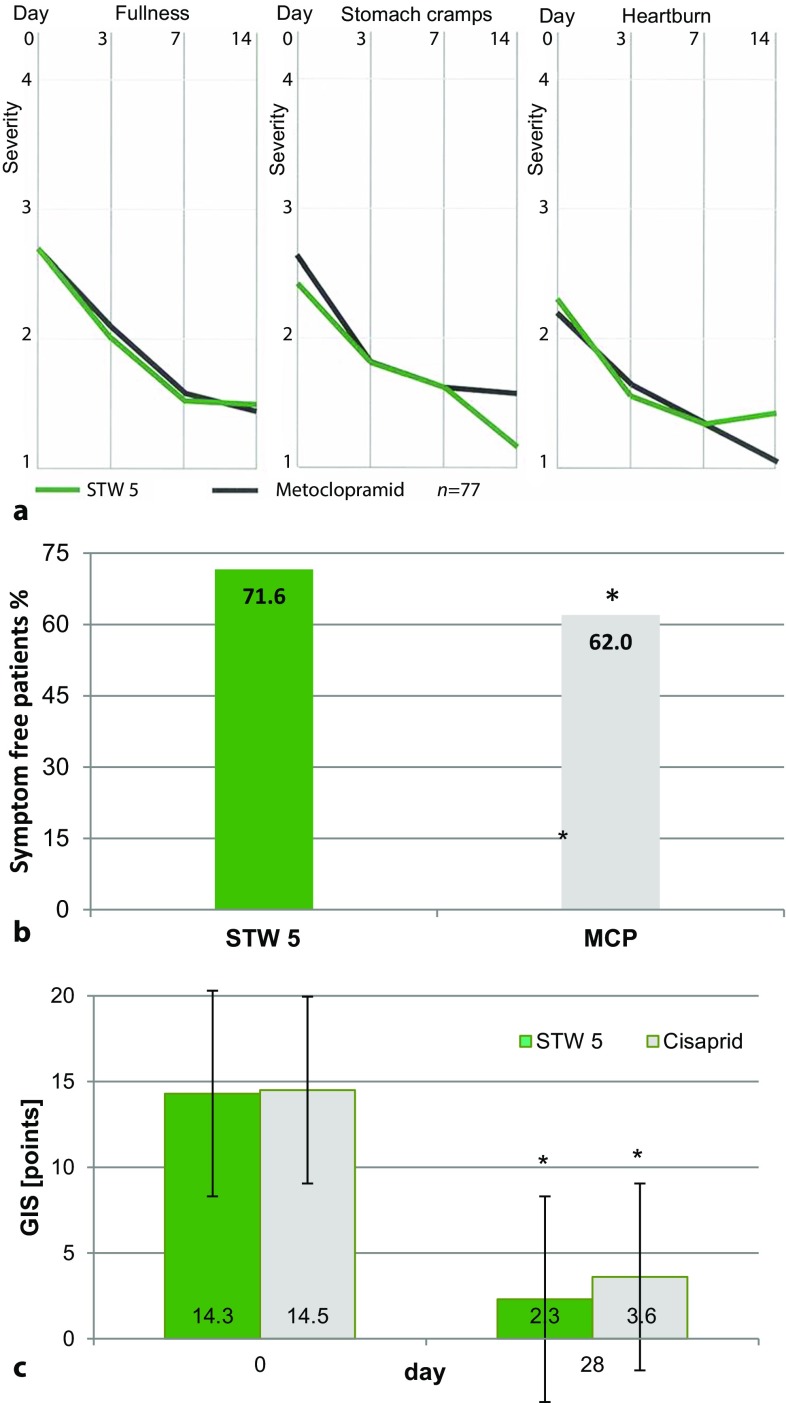
Comparison studies of STW 5 (Iberogast) and prokinetics. **a** Significant improvement of three gastrointestinal symptoms (*p* < 0.05) after treatment with STW 5 or metoclopramide (*MCP*) over 2 weeks in a single blind randomized clinical trial (*RCT*) [3]. **b** Significantly higher percentage of symptom-free patients with STW 5 vs. MCP (*p ≤ 0.05) in a retrospective surveillance study [4]. **c** Significant improvement of GIS (gastrointestinal symptom score) in a RCT (*p* < 0.001), demonstrating noninferiority of STW 5 vs. cisapride (*p ≤ 0.05; [6])

The first study, a single blind multicenter study with MCP liquid versus STW 5 was conducted in 77 patients with FGID, who complained at least three of the following symptoms: pressure or pain in the epigastrium, stomach cramps, feeling of fullness, eructation, nausea, urge to vomit, heartburn, or inappetence. Examination was conducted before starting therapy and on days 3, 7, and 14, and showed significant improvements of the validated gastrointestinal symptom score (GIS) against baseline in both groups. More than 50% of patients were free of symptoms after 14 days in both groups. There were no significant differences of clinical efficacy (Fig. 2), with a trend towards a lower number of adverse events in the STW 5 group [3].

In a second study, a retrolective, multicenter epidemiological postmarketing surveillance study, MCP and STW 5 were compared in routine clinical practice (*n* = 960) [4]. Before inclusion into analysis, patients should have suffered from at least three of ten symptoms of the GIS [5]. The main outcome variable was the improvement of GIS, changes of single symptoms, time until complete symptom relief, investigators’ judgement of efficacy and tolerability, duration of inability to work, and occurrence of adverse effects as secondary parameters. The proportion of symptom-free patients after treatment was significantly higher in STW 5 than MCP (72.6% vs. 62.8%) while the median treatment duration was almost equal. Also the extent of single symptom improvement and the median duration of inability to work were significantly different in favor of STW5, as well as the physician’s assessment of tolerability. Adverse drug reactions were documented only for MCP. The study confirmed the findings from prospective trials which showed STW 5 being effective and an appropriate alternative to the prokinetics.

In a third study, a double blind, double dummy randomized controlled clinical trial (RCTs) of high quality (Table 1), STW 5 and cisapride were compared in patients with dysmotility type of functional dyspepsia (61 patients per group). The primary endpoint was the improvement of the GIS after 4 weeks; efficacy and tolerability assessments, recurrence and safety parameters were the secondary endpoints. In all, 43 (STW 5) and 45 (cisapride) patients were included in the confirmatory analysis which showed noninferiority for STW 5 and no significant differences for the secondary endpoints, while there was a trend towards superior tolerability of STW 5. Thus, STW 5 showed comparability with cisapride [6] and indirectly also to MCP, because the comparable efficacy of MCP and cisapride in FGID has been proven in a high quality study [7].

The literature search for studies on the efficacy of STW 5 in IBS, revealed a double blind RCT [8, 9]. STW 5 (*n* = 51) and placebo (*n* = 52) were compared over 4 weeks. The main outcome variables were the change in total abdominal pain and the IBS symptom score. STW 5 was significantly superior to placebo in reducing the total abdominal pain score and the IBS symptom score after 4 weeks, likewise in the diarrhea or constipation dominant or alternating type of IBS. These results are supported by a non-interventional study (NIS) [9].

The search for further efficacy and safety data for STW 5 identified, among others, NIS and surveillances in more than 50,000 patients [10], also including children of all age groups [11, 12] and routine pharmacovigilance data from therapeutic use in roughly more than 70 million patients since its introduction to the market in 1960 [10, 13].

Accordingly, the safety profile of STW 5 is very benign [10, 13] and by far superior compared to that of MCP or domperidone. This can be shown by comparison of the relevant contents of the summaries of product characteristics (Table 2).

**Table 2:** Comparison of the field of application and the safety of STW 5 vs. metoclopramide and domperidone (oral dosage forms), as documented in summaries of product characteristics (SPCs, shortened) for Germany

Safety related sections from the SPC	STW 5	Metoclopramide	Domperidone
*Field of application*	For the treatment of functional and motility related gastrointestinal diseases such as functional dyspepsia and irritable bowel syndrome as well as for the supportive treatment of gastritis. These diseases manifest predominantly in complaints of stomach pain, feeling of fullness, bloating, gastro-intestinal cramps, nausea and heartburn	Prevention of delayed chemotherapy induced nausea and vomiting Prevention of radiotherapy induced nausea and vomiting Symptomatic treatment of nausea and vomiting, including acute migraine induced nausea and vomiting Metoclopramide can be used in combination with oral analgesics to improve the absorption of analgesics in acute migraine	For the treatment of the symptoms nausea and vomiting in adults and adolescents with an age of more than 12 years and a body weight of 35 kg at minimum
*Duration of use*	Basically, there is no restriction of the duration of use. The duration of use is determined by the form, severity and course of the disease	The maximum recommended duration of use is 5 days	As a rule, the maximum duration of use should not exceed one week
*Contraindication*	Hypersensitivity against the active substances Children below 3 years, due to the lack of sufficient data	Hypersensitivity against one of the constituents – Gastrointestinal bleeding, mechanical obstruction or gastrointestinal perforation, where a stimulation of motility is a risk – Phaeochromocytoma – Neuroleptic or metoclopramide triggered tardive dyskinesia – Epilepsy – Parkinson disease – Combination with levodopa or dopaminergic agonists – Known history of methamoglobinamia – Use in children below 1 year due to the enhanced risk of extrapyramidal diseases	– Hypersensitivity against one of the constituents – Prolactinoma – Disturbance of hepatic function – Prolonged QTc interval, electrolyte disturbances or congestive cardiac insufficiency – Concomitant use with drugs leading to prolonged QT, or with strong CYP3A4 inhibitors – Gastrointestinal bleedings, mechanical obstruction or gastrointestinal perforation where a stimulation of motility is a risk
*Special warnings and safety measures*	In case that symptoms persist or in case of lack of success of treatment for more than a week or in case symptoms worsen, a medical doctor should be consulted for excluding organic causes Generally, in children below 6 years, in case of abdominal pain a medical doctor should be consulted	*Neurological diseases:* Extrapyramidal diseases (usually reversible) Tardive dyskinesia (especially after prolonged use) Malign neuroleptic syndrome Symptoms of Parkinson disease can worsen. *Methemoglobinemia* *Cardiac diseases:* Care is needed in prolonged QTc interval, electrolyte disturbances (hypokalemia, hyperkalemia, hypomagnesemia) or congestive cardiac insufficiency, as well as with concomitant use with drugs leading to prolonged QT time *Functional disturbances of liver and kidney:* Reduction of dose needed	*Functional disturbances of kidney:* Reduce dosing frequency and dose *Cardiovascular effects:* Very rarely: Prolongation of QT interval and torsade de pointes in predisposed patients Enhanced risk of severe ventricular arrhythmias or sudden cardiac death, predominantly in high doses and predisposed patients with prolonged QTc interval, electrolyte disturbances (hypokalemia, hyperkalemia, hypomagnesemia) or congestive cardiac insufficiency, as well as with concomitant use of drugs leading to prolonged QT time Concomitant use with Levodopa can lead to increased plasma concentrations of levodopa
*Interactions with other drugs and other interactions*	None known	*Contraindicated combinations:* Concomitant use of levodopa or dopaminergic agonists *Combination to be avoided* Ethanol *Take care when combining with* Anticholinergic drugs, morphine derivatives, anxiolytics, sedative H1 antihistaminics, antidepressants, barbiturates, clonidine, neuroleptics, serotonergic drugs, digoxin, ciclosporin, mivacurium and suxamethonium, strong CYP2D6 inhibitors	*Combinations to be avoided:* Concomitant use of antacids or antisecretory drugs Concomitant use with drugs which are metabolized via CYP3A4 (e. g., ketoconazole, erythromycine) leads to enhanced plasma levels of domperidone *Contraindicated combinations:* Drugs leading to prolonged QT times – Antiarrhythmics class IA (e. g., disopyramid, hydrochinidine, chinidine) – Antiarrhythmics class III (e. g. amiodarone, dofetilide, dronedaron, ibutilide, sotalo). – Certain antipsychotics (e. g., haloperidol, pimozide, sertindole) – Certain antidepressants (e. g., citalopram, escitalopram) – Certain antibiotics (e. g., erythromycine, levofloxacine, moxifloxacine, spiramycine) – Certain antimycotics (e. g., pentamidine) – Certain antimalaria drugs (especially halofantrine, lumefantrine) – Certain gastrointestinal drugs (e. g., cisapride, dolasetrone, prucalopride) – Certain antihistaminics (e. g., mequitazine, mizolastine) – Certain anticancer drugs (e. g., toremifen, vandetanib, vincamin) – Certain other drugs (e. g., pepridil, diphemanil, methadone) Strong CYP3A4 inhibitors, e. g., – Protease inhibitors – Systemic azole antimycotics – Some macrolides (erythromycine, clarithromycine, telithromycine) *Combinations not recommended* Moderate CYP3A4 inhibitors (e. g. diltiazem, verapamil, makrolides) *Take care when combining with* – Drugs inducing bradycardia or hypopotassemia as well as the following macrolides: azithromycine, roxithromycine – Ketoconazole (prolongation of QTc, interactions) – Levodopa (interaction)
Impairment of the ability to drive and to use machines	None	Somnolence, drowsiness, dizziness, dyskinesias and dystonias impairing the ability to drive and to use machines	None or only negligible influence
Adverse events			
Very frequent	**–**	– Somnolence	**–**
Frequent	**–**	– Diarrhea – Asthenia – Extrapyramidal diseases (especially in children and young adults and in case of overdose), parkinsonism, akathisia – Depression – Hypertension	– Dryness of mouth
Occasionally	**–**	– Bradycardia – Amenorrhea, hyperprolactinemia – Hypersensitivity – Dystonia, dyskinesia, impaired consciousness – Hallucination – Confusion	– Anxiety, loss of libido – Somnolence, headache – Diarrhea – Exanthema, pruritus – Galactorrhea, breast pain and -tension – Asthenia
Rare	**–**	– Galactorrhea – Cramps	–
Very rare	– Hypersensitivity reactions (as e. g., exanthema, pruritus, dyspnea)	–	–
Unknown	**–**	– Methemoglobinemia, sulfhemoglobinemia – Cardiac arrest, atrioventricular blockage, QT prolongation, torsade de pointes – Gynecomastia – Anaphylactic reaction (including anaphylactic shock) – Tardive dyskinesia, which may be irreversible, malign neuroleptic syndrome – Acute hypertension in patients with phaeochromocytoma	– Allergic hypersensitivity (including anaphylactic shock) – Agitation, nervousness – Oculogyric crisis – Ventricular arrhythmias, prolongation of QTc time, torsade de pointes, sudden cardiac death – Urticaria, angioedema – Urine retention – Gynecomastia, amenorrhea – Abnormal liver function tests, hyperprolactinemia – Acathisia – Depression, – Extrapyramidal side effects, cramps, agitation (mainly in children)
Overdose	The acute oral toxicity studies in different animal species and long standing therapeutic experience in patients did not give hints on intoxications	Extrapyramidal diseases, somnolence, confusion, hallucination, cardiac and respiratory arrest	Symptoms of overdose were mainly observed in children: Agitation, change of consciousness, cramps, disorientation, somnolence, extrapyramidal reactions

Up to 2014 the fields of application of MCP included in addition motility disturbances of the upper gastrointestinal tract (e. g., in functional dyspepsia, heartburn, reflux esophagitis, functional pyloric stenosis), those of domperidone epigastic feeling of fullness and upper abdominal discomfort

Frequency of adverse events is classified as follows: *Very frequent* *≥1/10; Frequent* *≥1/100 to <1/10; Occasionally ≥1/1000 to <1/100; Rare ≥1/10,000 to <1/1000; Very rare <1/10,000; Unknown: can not be estimated based on the data available*

## Pharmacological mechanisms – multitarget action

The prokinetic MCP was shown to possess agonistic activities at serotonergic 5‑HT4 and muscarinic acetylcholine (ACh) receptors as well as antagonistic effects at dopaminergic D2 and 5‑HT3 receptors, while domperidone shows antagonistic activities at D2 receptors. Cisapride, on the other hand, is agonistic at the 5‑HT4 receptor, resulting in a tonificating prokinetic effect [14–16].

**Fig. 3:**
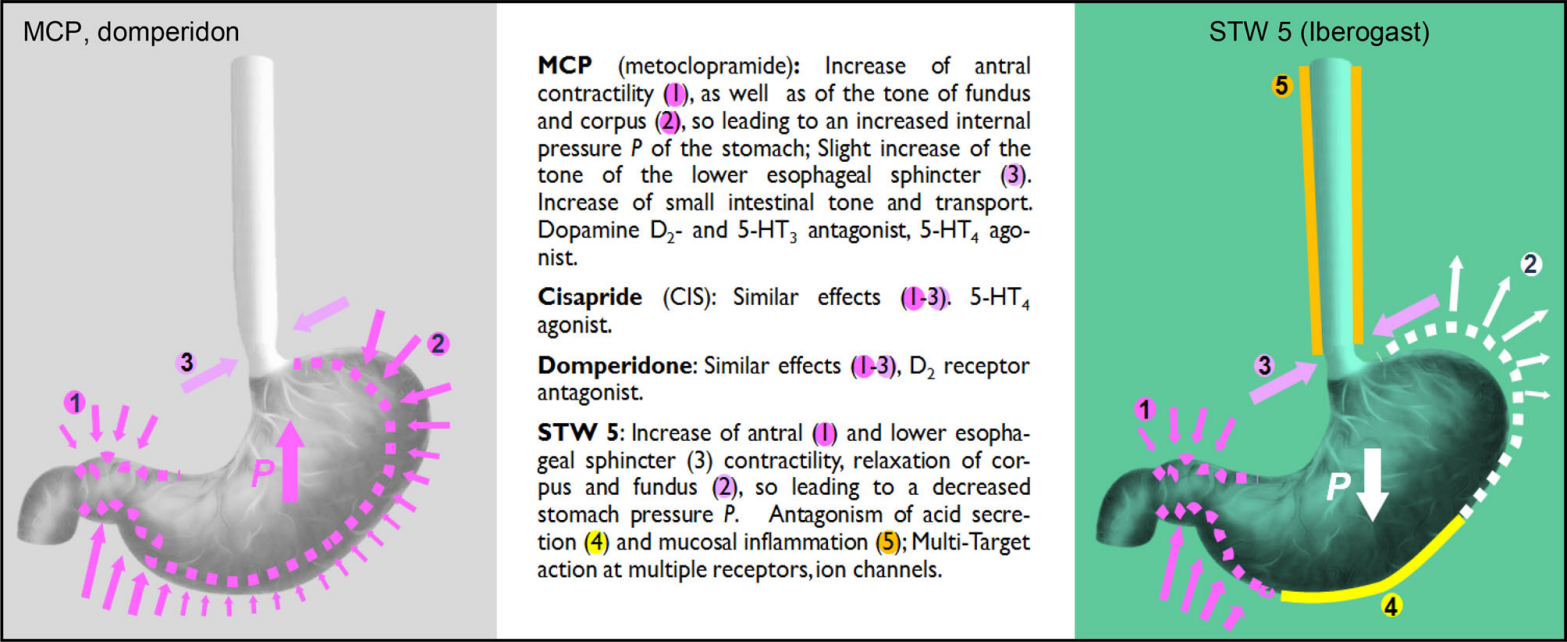
Pharmacological mechanisms of action of prokinetics (metoclopramide, domperidone, cisapride) and STW 5 [14–17, 21, 30]

For STW 5, several mechanisms (Fig. 3) have been identified in preclinical studies [17]. The primary studies [18] identified a dual mechanism of action on gastrointestinal motility, with a spasmolytic effect on ACh-induced contractions and a tonificating effect in the relaxed state. This has been confirmed in different pharmacological models [19, 20] and in human isolated intestinal segments [21] as well as in inflamed intestinal tissue in vitro and in vivo [22–24].

In the stomach, a region specific action was reported in vitro, based on an inhibition of Ca^2+^ influx via store-operated channels (SOC) in the gastric fundus and on a stimulation of Ca^2+ ^influx via L‑type Ca^2+ ^channels in the antrum [25]. The rapid and region-specific action has been confirmed in a human study in vivo [26]. In the lower esophageal sphincter, a tonificating action mediated by L‑type Ca^2+^channels was identified in vitro [21]. The components of the herbal combination STW 5 have been shown to act synergistically in pharmacological studies [27].

## Discussion

In recent years, for the prokinetics, despite of their apparently selective and specific action on receptors relevant for gastrointestinal function, severe central nervous or cardiac side effects have shown up. The safety profile of herbal medicinal products with their broad spectrum of pharmacologically active constituents obviously is by far superior. The question whether they are also equally effective and therefore a suitable substitute for the prokinetics in both FD and IBS may be answered positively at least for STW 5 considering the clinical studies comparing the effects of STW 5 versus MCP and cisapride, the proven efficacy in both FD and IBS, and the lack of severe side effects demonstrated in interventional and noninterventional studies and also in broad routine clinical use. Altogether, efficacy of STW 5 has been documented according to the recommendations of evidence-based medicine [28, 29]. The pharmacological data, addressing multiple pharmacological mechanisms relevant in the etiology of FGID, indicate a multitarget action, supplying a valid explanation for the clinical efficacy.

## Conclusion

The clinical studies comparing STW 5 with cisapride and MCP, and further clinical studies on FD and IBS clearly demonstrate its efficacy in FGID. The clinical data qualify STW 5 as a substitute for the prokinetics, which are no longer authorised for the treatment of FGID. In vitro and in vivo as well as human studies demonstrate spasmolytic as well as tonificating, prokinetic and anti-inflammatory effects which are in line with the reported clinical outcomes. The effects described are based on the multitarget action of the multitude of constituents of STW 5. In contrast to the prokinetics, which, despite of their apparent selectivity, have turned out to have specific severe adverse effects in rare cases, STW 5 has a favorable safety profile.

Thus, for the treatment of FGID, STW 5 was identified as a treatment with a clinical efficacy comparable to prokinetics, especially MCP and cisapride, but with a superior safety profile and without limitations of the duration of treatment. It presents an effective and safe treatment option for patients with both FD and IBS.
